# An *rfaH* Mutant of *Salmonella enterica* Serovar Typhimurium is Attenuated in Swine and Reduces Intestinal Colonization, Fecal Shedding, and Disease Severity Due to Virulent *Salmonella* Typhimurium

**DOI:** 10.3389/fvets.2014.00009

**Published:** 2014-10-09

**Authors:** Bradley L. Bearson, Shawn M. D. Bearson, Jalusa D. Kich, In Soo Lee

**Affiliations:** ^1^USDA, ARS, National Laboratory for Agriculture and the Environment, Ames, IA, USA; ^2^USDA, ARS, National Animal Disease Center, Ames, IA, USA; ^3^Embrapa Swine and Poultry, Concórdia, Santa Catarina, Brazil; ^4^Department of Biological Sciences and Biotechnology, Hannam University, Daejeon, South Korea

**Keywords:** *Salmonella*, vaccine, DIVA, rfaH, swine

## Abstract

Swine are often asymptomatic carriers of *Salmonella* spp., and interventions are needed to limit colonization of swine to enhance food safety and reduce environmental contamination. We evaluated the attenuation and potential vaccine use in pigs of a *Salmonella enterica* serovar Typhimurium mutant of *rfaH*, the gene encoding the RfaH antiterminator that prevents premature termination of long mRNA transcripts. Pigs inoculated with wild-type *S*. Typhimurium exhibited a significant elevation in average body temperature (fever) at 1 and 2 days post-inoculation; *rfaH*-inoculated pigs did not (*n* = 5/group). During the 7-day trial, a significant reduction of *Salmonella* in the feces, tonsils, and cecum were observed in the *rfaH*-inoculated pigs compared to wild-type inoculated pigs. To determine whether vaccination with the *rfaH* mutant could provide protection against wild-type *S*. Typhimurium challenge, two groups of pigs (*n* = 14/group) were intranasally inoculated with either the *rfaH* mutant or a PBS placebo at 6 and 8 weeks of age and challenged with the parental, wild-type *S*. Typhimurium at 11 weeks of age. The average body temperature was significantly elevated in the mock-vaccinated pigs at 1 and 2 days post-challenge, but not in the *rfaH*-vaccinated pigs. Fecal shedding at 2 and 3 days post-challenge and colonization of intestinal tract tissues at 7 days post-challenge by wild-type *S*. Typhimurium was significantly reduced in the *rfaH*-vaccinated pigs compared to mock-vaccinated pigs. Serological analysis using the IDEXX HerdChek Swine *Salmonella* Test Kit indicated that vaccination with the *rfaH* mutant did not stimulate an immune response against LPS. These results indicate that vaccination of swine with the attenuated *rfaH* mutant confers protection against challenge with virulent *S*. Typhimurium but does not interfere with herd level monitoring for *Salmonella* spp., thereby allowing for differentiation of infected from vaccinated animals (DIVA).

## Introduction

An estimated one million cases of foodborne illness are attributed to non-typhoidal *Salmonella* spp. each year in the U.S. at a predicted cost of $2.3 billion (1, 2). Non-typhoidal *Salmonella* spp. are a leading cause of hospitalization (35%) and death (28%) in the U.S. due to foodborne disease (2). Of 14 foodborne pathogens that cause 95% of foodborne illnesses, hospitalizations, and death in the U.S., non-typhoidal *Salmonella* spp. are the leading cause of disease burden (27%) measured by quality-adjusted life years (QALYs) (3).

Most of the >2,400 *Salmonella* serovars are ubiquitous in the environment and many can colonize food producing animals and poultry as well as wild animals and birds without causing overt disease. In the U.S., ~68,000 cases of pork-associated, human salmonellosis occur each year with an estimated social cost of $218 million annually (4). Of 104 pathogen–food combinations, the annual disease burden due to *S. enterica* in pork is estimated as the 13th highest. The most recent study from the USDA’s National Animal Health Monitoring System, Swine 2006, reported that 52.6% of swine production sites (representing 94% of the U.S. swine inventory) were positive for *Salmonella* spp. (5). Swine that are *Salmonella* carriers are a food safety risk for consumers of pork, an animal health risk to non-colonized/uninfected pigs, an economical risk to producers due to reduced feed conversion/efficiency (6, 7), and an environmental risk due to fecal shedding of the pathogen into swine manure that is used as a soil amendment. Interventions are needed to limit the colonization of swine with *Salmonella* spp. and reduce the risks to public health, animal health, and the environment.

The potential use of a *Salmonella enterica* serovar Typhimurium *rfaH* mutant as a vaccine candidate has been described (8, 9). The RfaH antiterminator prevents the premature termination of long mRNA transcripts encoded by large operons. A 5′ *cis*-acting operon polarity suppressor (*ops*) element is required to enhance transcription elongation from operons in the RfaH regulon (10, 11). *S*. Typhimurium *rfaH* mutants have decreased transcription of genes encoding lipopolysaccharide (LPS) core, O-antigen, *Salmonella* Pathogenicity Island 4 (SPI-4), flagellum/chemotaxis, and type III secretion system 1 (T3SS-1) (12). However, the decreased transcription of some of these operons may be due indirectly to the loss of LPS. A *S*. Typhimurium SL1344 *rfaH* mutant is attenuated for virulence (both oral and intraperitoneal inoculation) in the BALB/c mouse model of systemic disease compared to wild-type SL1344 (8). Immunization of BALB/c mice with three doses of an *rfaH* mutant [5 × 10^7^ colony forming units (CFU)] at 2-week intervals was protective against challenge with 5 × 10^7^ CFU of wild-type SL1344 at 2 weeks following the final vaccine dose (0/4 vs. 4/4 deaths for vaccinated and naïve mice). Further investigation indicated that vaccination of BALB/c mice with three increasing doses (2 × 10^7^, 2 × 10^8^, and 2 × 10^9^ CFU) of the SL1344 *rfaH* mutant at 2-week intervals resulted in significant protection against homologous (86%) and heterologous (40%) challenge with 5 × 10^6^ CFU of either wild-type SL1344 or wild-type *S*. Enteritidis NCTC13349, respectively (13). These results indicate that vaccination of mice with the SL1344 *rfaH* mutant provides some cross-protection against other serovars in addition to *S*. Typhimurium.

In the current study, an *rfaH* knockout mutant of *Salmonella enterica* serovar Typhimurium was constructed to determine whether: (1) the strain was attenuated in pigs, (2) would protect swine against virulent *S*. Typhimurium challenge, while (3) allowing for DIVA using the IDEXX HerdChek Swine *Salmonella* Test Kit. This is the first report of an *rfaH* mutant evaluation in swine and our results indicate that *rfaH*-vaccinated pigs have reduced disease severity, pathogen fecal shedding, and intestinal colonization due to virulent *S*. Typhimurium challenge, but swine vaccination did not interfere with the herd level monitoring system for *Salmonella* spp.

## Materials and Methods

### Strain construction and growth conditions

The *S*. Typhimurium BBS 202 (*rfaH*:*neo*) strain was constructed by recombineering as previously described (14). Briefly, oligos oBBI 189 (**atgcaatcctggtatttactgtactgcaaacgcgggcaacttca****gc**atagagcagtgacgtagtcgc) and oBBI 190 (**ctaaatcttgcgaaaaccggtgttttttacgctctgcttcactt****c**gatagctgaatgagtgacgtgc) were used to PCR amplify the oBBI 92/93-*neo* template for synthesis of a linear knockout fragment. The 5′ end (bold) of oBBI 189 and oBBI 190 have homology to the 5′ and 3′ *rfaH* target sequences (respectively) whereas the 3′ end (underlined) of the oligos have homology to oBBI 92/93-*neo* encoding an antibiotic resistance gene flanked by universal sequences that truncate potential translation of the target gene. Following PCR amplification, gel electrophoresis was performed using a 1% agarose gel in TBE and the oBBI 189/190-*neo* knockout fragment was gel purified using a BioRad Freeze ‘N Squeeze column (Hercules, CA, USA). The linear knockout fragment containing the *neo* gene was electroporated into arabinose-induced competent cells of *S*. Typhimurium BSX 7 (14) containing the plasmid pKD46 encoding Lambda *exo*, *bet*, and *gam* to facilitate recombination of the DNA fragment (15). Kanamycin-resistant transformants were screened by PCR to confirm the replacement of *rfaH* with the *neo* gene and this strain was stocked as BBS 195. Due to the *rfaH* mutant being a poor recipient for P22 binding, the *rfaH* gene was cloned into a plasmid to complement the *rfaH* mutation using primers oBBI 193 (caggaagacgcgttaaatcg) and oBBI 194 (gaacgatcgctaaatcttgc). The oBBI 193 primer binds within *yigC* (STM3978), ~625 bp upstream of the codon for the *rfaH* start methionine. The binding site for the oBBI 194 primer overlaps with the *rfaH* stop codon. Primers oBBI 193 and oBBI 194 were used to amplify the *rfaH* region from the *S*. Typhimurium χ4232 genome, and the PCR product was cloned using the pBAD TOPO TA Expression Kit (Invitrogen, Carlsbad, CA, USA) followed by transformation into One Shot TOP10 competent *E. coli*. The pBAD-oBBI193/194 plasmid was extracted from the TOP10 strain and transformed into BBS 195 (*rfaH*:*neo*), single colony isolated, and stocked as BBS 201. Using a P22 high-transducing phage lysate grown on BBS 201, the *rfaH*:*neo* mutation was transferred to wild-type *S*. Typhimurium χ4232, creating BBS 202.

The *S*. Typhimurium strains χ4232 and BBS 202 were grown in LB in the presence of nalidixic acid without and with kanamycin, respectively. Antibiotics were used at the following concentrations: ampicillin (100 μg/ml) for pKD46, kanamycin (50 μg/ml), and nalidixic acid (30 μg/ml).

### Ethics statement

All experimental procedures involving animals were in compliance with the recommended principles described in the Guide for the Care and Use of Laboratory Animals by the National Research Council of the National Academies and were approved by the USDA-ARS, National Animal Disease Center, Animal Care, and Use Committee.

### Animal trial #1

Ten crossbred, conventionally raised piglets from five *Salmonella*-fecal-negative sows were weaned at 12 days of age and shipped to the National Animal Disease Center, Ames, IA, USA. Siblings from each litter were divided and raised in two isolation rooms (*n* = 5/room). Piglets tested fecal-negative for *Salmonella* spp. thrice over a 4-week period using bacteriological culture with selective enrichment (16). At 6 weeks of age (day zero), pigs received an intranasal inoculation of 1 ml PBS containing either 1 × 10^9^ CFU of BBS 202 (*rfaH*:*neo*; *n* = 5) or *S*. Typhimurium χ4232 (wild-type; *n* = 5). Clinical symptoms and pathogen fecal shedding were monitored at 0, 1, 2, 3, 5, and 7 days post-inoculation (dpi) (see below).

### Animal trial #2

Twenty-eight crossbred, conventionally raised piglets from four *Salmonella*-fecal-negative sows were weaned at 12 days of age and shipped to the National Animal Disease Center, Ames, IA, USA. Equally divided from each litter, the pigs were raised in two isolation rooms (*n* = 14/room) and tested fecal-negative for *Salmonella* spp. twice over a 4-week period using bacteriological culture with selective enrichment. At 6 weeks of age, pigs received a 1 ml intranasal inoculation of either 1 × 10^9^ BBS 202 or a placebo (PBS); a booster inoculation occurred 2 weeks later at 8 weeks of age. At 11 weeks of age, all pigs were intranasally challenged with PBS containing 1 × 10^8^ CFU of wild-type *S*. Typhimurium (χ4232) and monitored for clinical disease symptoms, *Salmonella*-fecal shedding and intestinal colonization over a 7-day period (see below).

### Sample collection and processing

Swine body temperatures were determined using a rectal thermometer, and blood samples were obtained via the jugular vein at each inoculation time point as well as 7 days post-challenge. Fecal samples were obtained for quantitative and qualitative *Salmonella* culture analysis as previously described (16) with the addition of XLT-4 medium containing the appropriate antibiotics to select for each strain (nalidixic acid for χ4232; nalidixic acid and kanamycin for BBS 202). At 7 dpi, all pigs were euthanized and necropsies performed to obtain tissue samples from the tonsil and the intestinal tract (ileal Peyer’s Patches, ileocecal lymph nodes, and cecum) for quantitative and qualitative *Salmonella* culture analysis as previously described with slight modification (17). One hundred microliters of each homogenized tissue (1 g of tissue combined with 2 ml of PBS) was aliquoted onto BGS and XLT-4 media containing kanamycin and/or nalidixic acid (see below).

To differentiate between the wild-type and *rfaH* mutant strains in the bacteriological culture assays, fecal and tissues samples were plated on medium containing nalidixic acid alone and medium containing both nalidixic acid and kanamycin. As both χ4232 and BBS 202 are resistant to nalidixic acid, the medium with nalidixic acid and kanamycin was used to identify BBS 202. Subtraction of the number of colonies present on the kanamycin–nalidixic acid medium from the colonies present on the nalidixic acid only medium determined the number of χ4232 colonies.

All statistical analyses were performed in GraphPad Prism 5.01 (GraphPad Software, Inc., La Jolla, CA, USA). Statistical analysis of body temperatures for each treatment group was performed using repeated measures ANOVA followed by Bonferroni’s Multiple Comparison Test. Statistical analysis of *Salmonella* present (Log_10_ colony forming units per gram) in fecal samples was analyzed by two-way ANOVA followed by Bonferroni post-tests. Statistical analysis of *Salmonella* present (Log_10_ colony forming units per gram) in tissues at 7 dpi was analyzed using the Two Sample *t*-test for the Means.

### Fecal score analysis

Each fecal sample in animal trial #2 was assigned a diarrhea score (18) by the same four evaluators at the time of collection (prior to information concerning shedding status) using a scale of 1–5 (1 = dry feces, 2 = moist feces, 3 = mild diarrhea, 4 = severe diarrhea, and 5 = watery diarrhea). Statistical analysis of diarrhea scores was analyzed by two-way ANOVA followed by Bonferroni post-tests.

### IDEXX HerdChek swine *Salmonella* antibody assay

Serum antibody analysis to LPS antigen derived from *Salmonella* serogroups B, C1, and D was performed using the IDEXX HerdChek Swine *Salmonella* Test Kit (IDEXX Europe B.V., Hoofddorp, Netherlands). The assay and interpretation were performed per the manufacturer’s instructions. Briefly, the 30-min incubation was performed and the sample to positive (S/P) ratio was calculated for each sample using S/P = (sample mean − negative control mean)/positive control mean − negative control mean). A sample’s S/P ratio of ≥0.25 was considered positive and <0.25 was considered negative. Analysis of piglet serum was performed in-house whereas sow screening was performed by Boehringer Ingelheim, Vetmedica, Inc. (Ames, IA, USA).

## Results and Discussion

### *rfaH* mutant of *S*. Typhimurium is attenuated in swine

**Figure 1:**
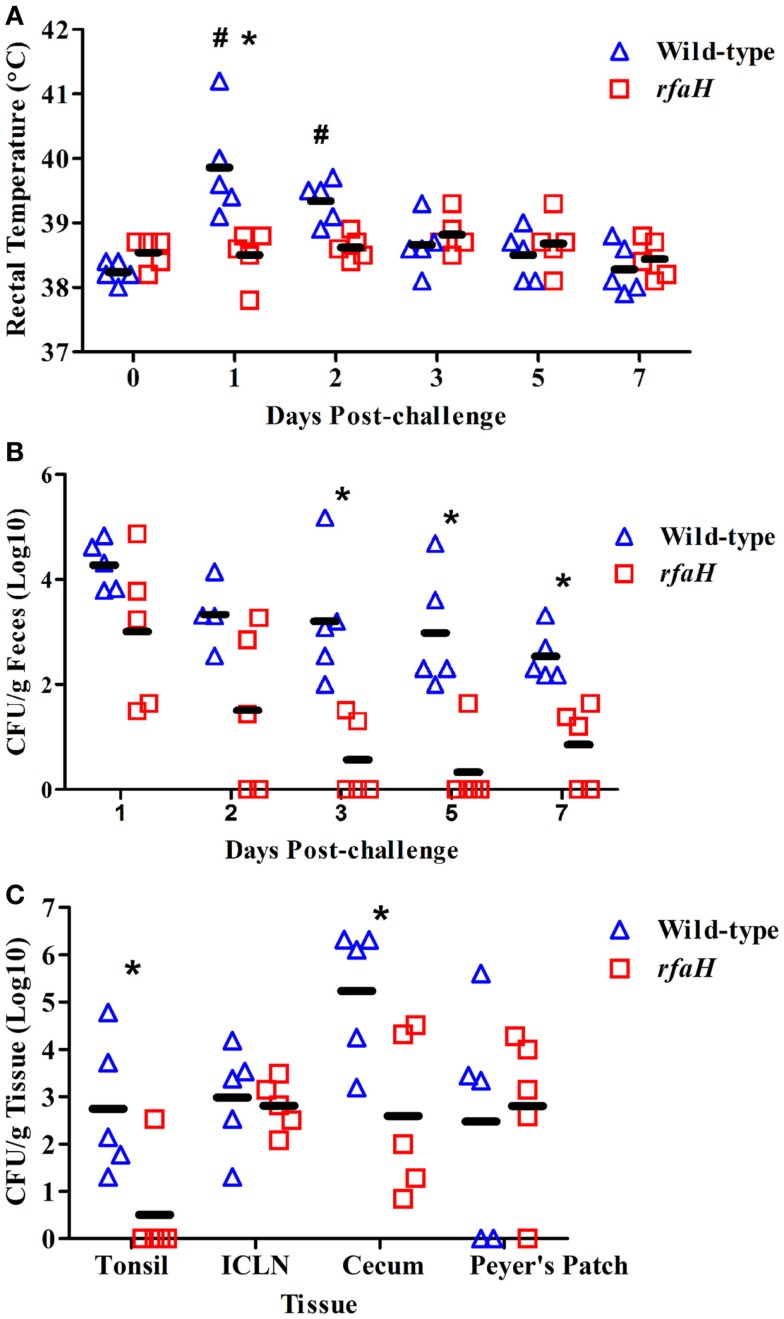
**Average rectal temperature, fecal shedding, and tissue colonization were significantly reduced in pigs inoculated with the *rfaH* mutant compared to the parental wild-type *S*. Typhimurium**. At 6 weeks of age, swine were inoculated with 1 ml of PBS containing either 1 × 10^9^ colony forming units (CFU) of BBS 202 (*rfaH*:*neo*; *n* = 5) or *S*. Typhimurium χ4232 (wild-type; *n* = 5). **(A)** Swine rectal temperature at 0, 1, 2, 3, 5, and 7 dpi. **(B)** *Salmonella* bacteriological analysis (colony forming units per gram) of swine fecal samples obtained at 1, 2, 3, 5, and 7 dpi. **(C)** *Salmonella* bacteriological analysis (colony forming units per gram) of the tonsil, ileocecal lymph nodes (ICLN), Peyer’s Patches, and cecum obtained during necropsy at 7 dpi. *Significant difference (*P* < 0.05) between swine inoculated with wild-type *S*. Typhimurium compared to the *rfaH* mutant at the indicated time point. ^#^Significant difference (*P* < 0.05) in wild-type inoculated pigs at the indicated time point compared to day 0.

An *rfaH* mutant of *S*. Typhimurium was constructed and tested for attenuation *in vivo*. Following a 1 × 10^9^ CFU inoculation, the average body temperature (fever) of pigs inoculated with the parental, wild-type *S*. Typhimurium χ4232 was significantly higher at day 1 (39.9°C) and day 2 (39.3°C) post-inoculation compared to day 0 (38.2°C) for the wild-type strain (Figure 1A; *P* < 0.05). Also, the average body temperature of pigs inoculated with wild-type *S*. Typhimurium was significantly higher at 1 dpi compared to pigs inoculated with the *rfaH* mutant (Figure 1A; 39.9°C vs. 38.5°C; *P* < 0.05). In fact, the average body temperature of the pigs inoculated with the *rfaH* mutant did not significantly increase throughout the 7-day trial. Swine fecal shedding of the *S*. Typhimurium *rfaH* mutant was significantly reduced (up to 2.5 logs) at days 3, 5, and 7 dpi compared to wild-type *S*. Typhimurium (Figure 1B; *P* < 0.05). Furthermore, tissue colonization was significantly lower in the tonsil (>2 logs) and cecum (2.5 logs) in the pigs inoculated with the *rfaH* mutant compared to wild-type *S*. Typhimurium (Figure 1C; *P* < 0.05), although no difference in colonization of the ileal Peyer’s Patches or ileocecal lymph nodes occurred. Thus, the *rfaH* mutant is attenuated for clinical symptoms (fever) with reduced fecal shedding and tissue colonization compared to the parental, wild-type *S*. Typhimurium.

### Vaccination of pigs with the *rfaH* mutant significantly reduces the effects of subsequent challenge with wild-type *S*. Typhimurium

**Figure 2:**
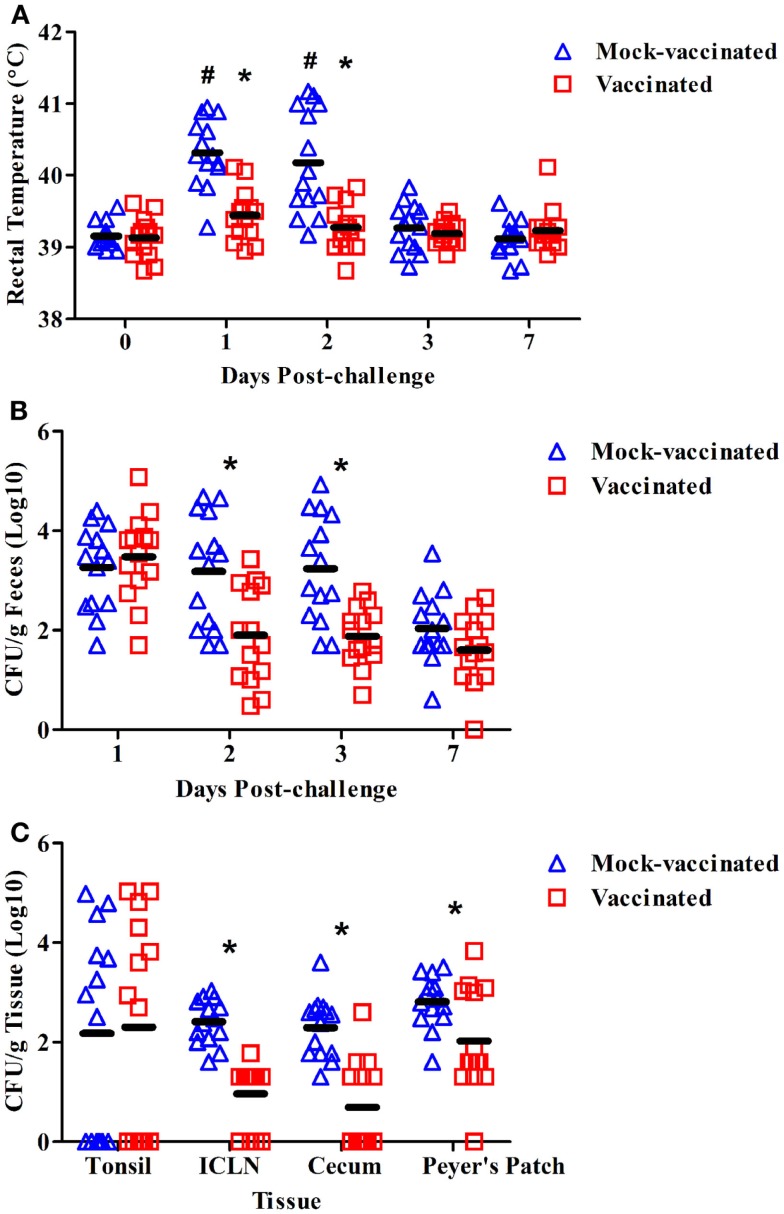
**The *rfaH*-vaccinated pigs had significantly lower average rectal temperature, fecal shedding, and tissue colonization compared to the mock-vaccinated pigs following challenge with wild-type *S*. Typhimurium**. At 6 and 8 weeks of age, pigs were inoculated with the potential *rfaH* vaccine strain (BBS 202) or mock–vaccinated with PBS (*n* = 14/group). At 11 weeks of age, all 28 pigs were challenged with 1 × 10^8^ CFU of the parental, wild-type *S*. Typhimurium χ4232. **(A)** Swine rectal temperature at 0, 1, 2, 3, and 7 days post-challenge. **(B)** *Salmonella* bacteriological analysis (colony forming units per gram) of swine fecal samples obtained at 1, 2, 3, and 7 days post-challenge. **(C)** *Salmonella* bacteriological analysis (colony forming units per gram) of the tonsil, ileocecal lymph nodes (ICLN), Peyer’s Patches, and cecum obtained during necropsy at 7 dpi. *Significant difference (*P* < 0.05) comparing *rfaH*-vaccinated to mock-vaccinated pigs at the same time point. ^#^Significant difference (*P* < 0.05) in mock-vaccinated pigs at the indicated time point compared to day 0.

To determine whether vaccination with the *rfaH* mutant could provide protection against virulent *S*. Typhimurium challenge in swine, pigs were inoculated with the potential vaccine strain at 6 weeks of age and boosted 2 weeks later. As similarly reported in the attenuation trial above, no significant increase in average body temperature was observed following vaccination or booster vaccination of the pigs, thereby corroborating the data presented in Figure 1A for the *rfaH* mutant (data not shown). Mock-vaccinated pigs (*n* = 14) and *rfaH*-vaccinated pigs (*n* = 14) were challenged with 1 × 10^8^ CFU of the parental, wild-type *S*. Typhimurium at 11 weeks of age. The swine rectal temperature increased significantly at days 1 (40.3°C) and 2 (40.1°C) post-challenge compared to day 0 (39.1°C) in the mock-vaccinated pigs (Figure 2A; *P* < 0.05). Moreover, the swine rectal temperature was significantly increased in mock-vaccinated pigs compared to *rfaH*-vaccinated pigs at days 1 (40.3°C vs. 39.4°C) and 2 (40.1°C vs. 39.3°C) post-challenge (Figure 2A; *P* < 0.05). Following challenge with wild-type *S*. Typhimurium, the average body temperature of the pigs vaccinated with the *rfaH* mutant did not significantly increase throughout the 7-day trial. The average diarrhea score, a subjective measurement of moisture content, was significantly higher in mock-vaccinated pigs compared to *rfaH*-vaccinated pigs at 2 dpi (2.7 vs. 2.1, respectively; *P* < 0.05). Swine fecal shedding of *S*. Typhimurium was significantly increased (~1.5 logs) at days 2 and 3 post-challenge in mock-vaccinated pigs compared to *rfaH*-vaccinated pigs (Figure 2B; *P* < 0.05). Furthermore, *S*. Typhimurium colonization was significantly decreased in the ileocecal lymph nodes, ileal Peyer’s Patches, and cecum (~10-fold) at 7 days post-challenge in *rfaH*-vaccinated pigs compared to mock-vaccinated pigs (Figure 2C; *P* < 0.05). Hence, vaccination with the *rfaH* mutant reduced clinical symptoms (fever and diarrhea), *Salmonella*-fecal shedding and intestinal colonization upon challenge with virulent *S*. Typhimurium.

### *rfaH* mutant is a DIVA vaccine

**Figure 3:**
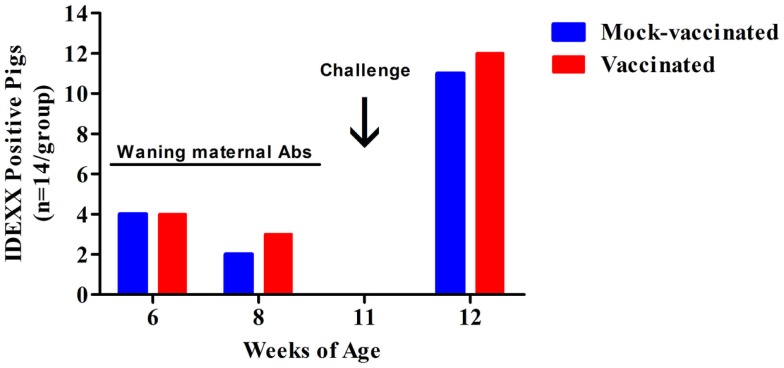
**Serum from *rfaH*-vaccinated swine is negative in the IDEXX HerdChek Swine *Salmonella* Test Kit 5-weeks after initial vaccination**. The number of pigs with serum containing antibodies to *Salmonella* LPS at 6-weeks (initial vaccination), 8-weeks (booster vaccination), 11-weeks (challenge), and 12-weeks (7 days post-challenge) of age. The number of positive pigs at each time point are shown. All pigs were challenged with wild-type *S*. Typhimurium at 11-weeks of age. A sample’s S/P ratio of ≥0.25 was considered positive and <0.25 was considered negative.

To determine if the pigs vaccinated with the *rfaH* mutant were producing detectable antibodies to *Salmonella* LPS antigen, sera from all 28 pigs (14 mock-vaccinated and 14 *rfaH*-vaccinated) were assayed using the IDEXX HerdChek Swine *Salmonella* Test Kit at multiple time points including 11-weeks of age (5 weeks following initial inoculation with the *rfaH* mutant and the day of wild-type *S*. Typhimurium challenge). All 28 pigs were negative for antibodies to *Salmonella* LPS antigen by ELISA at 11-weeks of age, indicating that vaccination with the *S*. Typhimurium *rfaH* mutant did not stimulate the production of antibodies against LPS (Figure 3). Therefore, the *rfaH* vaccine strain could be used in conjunction with *Salmonella* surveillance programs in swine for DIVA. At 12-weeks of age (7-days post-challenge with wild-type *S*. Typhimurium), the sera from 85% of *rfaH*-vaccinated and 78% of mock-vaccinated pigs were positive in the LPS ELISA assay, signifying that seroconversion to *Salmonella* LPS antigen following wild-type *S*. Typhimurium challenge had occurred. These results indicate that vaccination of swine with the *rfaH* DIVA vaccine does not interfere with the detection of a subsequent exposure to *Salmonella*.

At 6-weeks of age, 8 of 28 piglets (4 pigs in each of the two treatment groups) were positive for antibodies to *Salmonella* LPS antigen by ELISA (Figure 3). These eight piglets were siblings from the same sow and their antibodies waned as the age of the piglets increased. Taken together, these data indicate that the antibodies to *Salmonella* LPS in these piglets were maternal antibodies. *Salmonella* surveillance data including Swine 2006 suggests that *Salmonella* colonization in swine herds is not unusual. From 2011 to 2013, we screened 112 sows from 4 commercial swine facilities for the presence of *Salmonella* spp. in feces and antibodies to *Salmonella* LPS in serum. *Salmonella* was isolated from the feces of 24 sows (21%) and antibodies to *Salmonella* LPS were detected in the serum of 107 sows (95%). This indicates that although not all are actively shedding *Salmonella*, 95% of sows had a previous exposure to *Salmonella* spp. Therefore, our finding that a subset of the piglets in this vaccine trial had maternally acquired antibodies to *Salmonella* LPS is not surprising. Furthermore, since 95% of the sows tested were *Salmonella* antibody positive, exposure of pigs to *Salmonella* on-farm is probable, indicating a need for improved interventions including vaccination.

Previous investigations have described potential DIVA vaccines for swine utilizing different *Salmonella* gene targets (19–21). A limitation of some currently licensed *Salmonella* vaccines is that vaccination produces *Salmonella* antibodies in the host that cannot be differentiated from antibodies produced during an active infection, and therefore could interfere with surveillance programs that monitor *Salmonella* in swine production facilities. To eliminate potential interference, a licensed vaccine (Salmoporc, IDT Biologika GmbH) was modified (Δ*ompD*) and a new ELISA was developed to distinguish between infected and vaccinated animals (19). The modified vaccine did not stimulate an immune response that was detected in their anti-OmpD ELISA. However, current surveillance programs (ongoing since the early 1990s) utilize a *Salmonella* LPS ELISA, and switching to another ELISA method would need further validation prior to implementation in swine production. The use of *S*. Typhimurium LPS mutants for DIVA vaccine development has also been explored (20). Specifically, *S*. Typhimurium Δ*rfaJ* and Δ*rfaL* mutants when inoculated into swine did not stimulate an anti-*Salmonella* LPS response in contrast to the parental wild-type *S*. Typhimurium. However, further experiments in pigs were not performed utilizing these specific *S*. Typhimurium (Δ*rfaJ* and Δ*rfaL*) mutants, although the Salmoporc vaccine strain (above) was modified to incorporate the Δ*rfaJ* mutation (21). Inoculation of pigs with the Salmoporc-Δ*rfaJ* strain did not induce *Salmonella*-specific antibodies to LPS when serum was analyzed using the IDEXX HerdChek Swine *Salmonella* Test. In a *S*. Typhimurium transmission experiment whereby vaccinated (Salmoporc-Δ*rfaJ*), non-challenged pigs were exposed to *S*. Typhimurium-challenged “seeder” pigs, vaccination did not significantly decrease the adjusted transmission ratio compared to non-vaccinated pigs. As we performed a challenge experiment and not a transmission experiment in this study, we cannot directly compare our results to those of De Ridder et al.

Investigations in BALB/c mice have previously demonstrated that a *S*. Typhimurium SL1344 *rfaH* mutant is attenuated and protective against challenge with wild-type *S*. Typhimurium SL1344 (8). Mutations in *rfaH* have also been constructed in *S*. Typhimurium UK-1 and *S*. Gallinarum 287/91, and virulence attenuation has been demonstrated for both of these *rfaH* mutants in BALB/c mice and Rhode Island Red chicks, respectively (9, 22). Furthermore, inoculation of Rhode Island Red chickens with two doses of the *S*. Gallinarum 287/91 *rfaH* mutant (χ11571) provided significant protection against challenge with *S*. Gallinarum χ4173. However, due to concerns by these investigators that *rfaH* mutants may not adequately stimulate a robust immune response (due to potentially poor colonization of the intestinal tract and reduced invasion of epithelial cells in the intestine), strains of *S*. Typhimurium UK-1 and *S*. Gallinarum 287/91 were also constructed with *rfaH* expression under the control of the arabinose promoter (P_BAD_), resulting in regulated delayed expression of *rfaH* (9, 22). The *S*. Typhimurium UK-1 ΔP_rfaH178_ mutant (χ9735) with regulated delayed expression of *rfaH* was attenuated ~100-fold compared to wild-type *S*. Typhimurium UK-1 but was not as attenuated as the *rfaH* knockout mutant (χ9445 Δ*rfaH49*) at >10,000-fold. In three-day old Rhode Island Red chicks, the *S*. Gallinarum 287/91 strain χ11386 (ΔP_rfaH178_) with regulated delayed expression of *rfaH* retained full virulence compared to the wild-type *S*. Gallinarum 287/91 parent. In contrast, the *S*. Gallinarum *rfaH* knockout mutant (χ11571) was attenuated ~1,000-fold. Although the mouse and chick experiments were performed with two different *Salmonella* serovars, the results using the regulated delayed expression of *rfaH* may highlight differences between animal models that may impact the efficiency of this delivery method. Clearly, the expression of *rfaH* following arabinose induction does not wane quick enough to attenuate the virulence of *S*. Gallinarum χ11386 (ΔP_rfaH178_) in Rhode Island Red chicks. Results from the *S*. Gallinarum *rfaH* (χ11571) vaccine trial in Rhode Island Red chickens and our *S*. Typhimurium *rfaH* vaccine trial in swine indicate that the initial concern that *rfaH* knockout mutants may be excessively attenuated and therefore not stimulate a sufficient immune response to provide protection against challenge may be unwarranted. Colonization of swine intestinal tissues at 7 dpi with the BBS 202 (*rfaH*) strain indicates that the strain is present in multiple pigs for immune stimulation.

Although the results of the vaccination trial presented in this study are encouraging, additional investigations may be proposed to further evaluate the use of an *rfaH* mutant as a potential vaccine candidate in swine. For example, in our pig trials, we typically euthanize the animals at 7 days post-challenge in order to quantitatively evaluate tissue colonization by *Salmonella* (after 7 d.p.i., quantitation in tissues steadily decreases resulting in only a qualitative assessment (±), especially with a 10^8^ inoculum). Therefore, persistence of the *rfaH* mutant or of the challenge strain in the vaccinated vs. mock-vaccinated pigs over an extended period of time was not determined. Other parameters to be evaluated in future studies include inoculation route, inoculum dose, and age for vaccination (based on efficacy and industry practices), vaccination protection against *Salmonella* transmission from a *Salmonella*-shedding pig to a naïve pig, as well as evaluation of the vaccine in a larger population of pigs. Despite the need for further investigation, this initial evaluation of an *rfaH* mutant in swine as a potential vaccine candidate addressed three objectives: attenuation, protection, and interference with *Salmonella* surveillance. Vaccination of swine with the attenuated *S*. Typhimurium *rfaH* vaccine strain (BBS 202) reduced *S*. Typhimurium intestinal colonization and fecal shedding but did not stimulate an anti-*Salmonella* LPS immune response that would compromise *Salmonella* surveillance programs for swine herds. Thus, vaccination with the *S*. Typhimurium *rfaH* vaccine strain permits the DIVA.

## Conflict of Interest Statement

Mention of trade names or commercial products in this article is solely for the purpose of providing specific information and does not imply recommendations or endorsement by the U.S. Department of Agriculture. USDA is an equal opportunity provider and employer.
